# An Integrated Microfluidic System for One-Stop Multiplexed Exosomal PD-L1 and MMP9 Automated Analysis with Deep Learning Model YOLO

**DOI:** 10.3390/mi16111208

**Published:** 2025-10-24

**Authors:** Yunxing Lu, Wenjing Zhang, Qiang Shi, Jianan Hui, Jieyu Wang, Yiman Song, Xiaoyue Yang

**Affiliations:** 1 School of Science and Technology, Shanghai Open University, Shanghai 200433, China; yunxinglu2025@163.com (Y.L.); shiqiang@sou.edu.cn (Q.S.); 2 Department of Obstetrics and Gynecology, The Second Affiliated Hospital of Soochow University, Suzhou 215000, China; zwj20190805@163.com; 3 State Key Laboratory of Transducer Technology, Shanghai Institute of Microsystem and Information Technology, Chinese Academy of Sciences, Shanghai 200050, China; jiananhui2@mail.sim.ac.cn (J.H.); wjywaka@foxmail.com (J.W.); yimansong2025@163.com (Y.S.); 4 Shanghai Frontier Innovation Research Institute, Shanghai 201108, China; 5 School of Stomatology, Dalian Medical University, Dalian 116044, China; 6 The International Peace Maternity and Child Health Hospital, School of Medicine, Shanghai Jiao Tong University, Shanghai 200030, China; 7 Shanghai Key Laboratory of Embryo Original Diseases, Shanghai 200030, China

**Keywords:** exosomes, integrated microfluidic system, deep learning, immune escape, physical invasion

## Abstract

While immune escape and physical invasion are two key pathways in tumor development, traditional methods for analyzing their exosomal markers are often complex and face identification bias. Microfluidic technology offers significant advantages for non-invasive liquid biopsy, particularly in the analysis of tumor progression markers carried by exosomes. Here, we developed an integrated microfluidic system for the sensitive, accurate, totally on-chip exosome isolation and automatic quantification of tumor progression markers PD-L1 and MMP9. This platform leverages microfluidic design principles for efficient sample mixing and monodisperses microbeads for precise analysis, allowing for complete processing within 40 min. The system’s high efficiency and precision are further enhanced by a lightweight YOLOv5-based positional migration strategy that automates fluorescence quantification. Validation using four different cell lines demonstrated distinct exosomal protein signatures with a low detection limit of 12.58 particles/μL. This innovative microfluidic chip provides a sensitive and easy-to-handle tool for exosomal marker analysis, holding great potential for cancer identification and personalized therapy guidance in the era of point-of-care testing (POCT).

## 1. Introduction

Exosomes are cell-derived, membrane-bound nanovesicles, typically ranging from 30 to 200 nm in diameter, that play a pivotal role in intercellular signaling. By transferring a diverse cargo of bioactive molecules, including proteins, lipids, and nucleic acids, they modulate physiological and pathological processes [1,2,3]. In the context of oncology, exosomes secreted by tumor cells are particularly significant. They carry molecular markers that are reflective of their parental cells and contribute to key oncogenic processes such as tumor progression, immune evasion, and the establishment of pre-metastatic niches [4,5,6,7]. Their inherent stability in biofluids and relative abundance make them exceptionally attractive biomarkers for liquid biopsy, offering a non-invasive advantage over traditional tissue biopsies and demonstrating potential superiority to other circulating markers like circulating tumor DNA (ctDNA) and circulating tumor cells (CTCs) [6,8,9].

Within the rich exosomal proteome, specific markers hold high clinical relevance. Programmed Death-Ligand 1 (PD-L1) is an immune checkpoint protein instrumental in tumor-mediated immunosuppression. When expressed on tumor cells or exosomes, PD-L1 binds to the PD-1 receptor on activated T-cells, inducing T-cell anergy or apoptosis and allowing the tumor to evade immune surveillance. Consequently, the level of exosomal PD-L1 (exoPD-L1) can serve as a systemic indicator of a tumor’s immunosuppressive activity and predict its potential response to immunotherapy. Concurrently, Matrix Metalloproteinase 9 (MMP9), a zinc-dependent endopeptidase, is critically involved in the degradation of the extracellular matrix (ECM), a process fundamental to tumor invasion and metastatic dissemination [10,11]. The functions of PD-L1 (immune escape) and MMP9 (physical invasion) represent two distinct yet complementary mechanisms of cancer progression [6,12,13]. A tumor’s aggressiveness is multidimensional, requiring it to both evade the host immune system and physically breach tissue barriers to spread. Measuring only one of these markers provides an incomplete portrait of the tumor’s phenotype. Therefore, multiplexed quantification of both PD-L1 and MMP9 on exosomes can provide a more holistic and functionally relevant snapshot of tumor biological state, offering superior diagnostic and prognostic power by capturing insights into both its immuno-suppressive capacity and its invasive potential [14,15,16].

Despite this promise, the clinical translation of exosome-based diagnostics is hampered by persistent challenges in their isolation and analysis [6,7,17,18]. Gold-standard techniques such as ultracentrifugation are not only costly and labor-intensive but also yield preparations with potential contaminants, including protein aggregates and other vesicles of similar size. Subsequent analytical methods, including Enzyme-Linked Immunosorbent Assay (ELISA) and western blotting, typically demand large sample volumes and involve multiple, protracted steps, rendering them unsuitable for rapid, point-of-care diagnostics [19,20]. To address these limitations, microfluidic technology has emerged as a powerful alternative, leveraging scale effects to enhance sensitivity while reducing reagent consumption and reaction times [21,22,23,24]. Various microfluidic strategies have been explored, from physical sorting using nanoscale lateral displacement (nano-DLD) arrays to immunoaffinity capture targeting common exosome surface antigens like CD63 or CD81 [25,26,27,28]. However, these methods can face their own obstacles, including complex fabrication protocols, susceptibility to channel clogging, and fluorescent signal interference from bead aggregation during quantification. In addition, the integration of deep learning has introduced a new dimension to microfluidic analysis, demonstrating immense potential in processing and interpreting complex data from high-throughput platforms [29,30,31]. Leveraging deep learning’s advanced capabilities for efficient image processing and information analysis, models can automatically identify and classify biomarkers from microscopic images or sensor signals, significantly enhancing detection accuracy and efficiency [32,33,34,35].

In this research, we present an integrated microfluidic platform for the rapid, multiplexed analysis of exosomal PD-L1 and MMP9. The system is engineered to perform the entire analytical workflow through a single chip, from sample preparation including reagent mixing, exosome isolation, and fluorescent labeling to automatic detection and calculation within 40 min. A Y-shape dual-inlet structure coupled with a serpentine channel for highly efficient on-chip mixing and immunoreaction coupling, followed with distinct detection arrays for single-bead-level signal identification. Additionally, the lightweight YOLOv5-based deep learning algorithm is employed with a positional migration strategy for automated, accurate, and unbiased quantification. We validated this platform by profiling exosomes from three cancer cell lines—HepG2 (hepatocellular carcinoma), SK-LU-1 (non-small cell lung cancer), and Cal-27 (oral squamous cell carcinoma) and control HUVEC and RPMI1640 to demonstrate its capacity for rapid and differential tumor profiling.

## 2. Materials and Methods

### 2.1. Reagents and Materials

Polydimethylsiloxane (PDMS, Sylgard 184) was obtained from Dow Corning Co. (Midland, MI, USA). The chemical reagents 1-ethyl-3-[3-dimethylaminopropyl] carbodiimide (EDC), Sulfo-N-hydroxysulfosuccinimide (NHS), and trichloro(1H,1H,2H,2H-perfluorooctyl) silane (PFOTS) were acquired from Sigma-Aldrich (St. Louis, MO, USA). Surfactant (Tween-20) and Phosphate-buffered saline (PBS) were products of Sangon Biotech. Bovine serum albumin (BSA) was purchased from Equitech-Bio-Inc. Carboxylic-functionalized magnetic beads (15 µm diameter) were sourced from BaseLine ChromTech Research Center (Tianjin, China). The following antibodies were used: anti-CD63 antibody (for capture, Abcam, Cambridge, UK), Fluorescence 488 labeled anti-PD-L1 antibody (for direct detection, Abcam, UK), mouse monoclonal anti-MMP9 antibody (primary antibody for indirect detection, Abcam, UK) and Alexa Fluor 488-conjugated goat anti-mouse IgG (H + L) secondary antibody (Thermo Fisher Scientific, Waltham, MA, USA). The cell lines HepG2, SK-LU-1, Cal-27 and HUVEC were procured from the Cell Bank of the Shanghai Institutes for Biological Sciences (Shanghai, China). Fetal bovine serum (FBS) and Roswell Park Memorial Medium (RPMI) 1640, MEM, DMEM were from Gibco Thermo Fisher Scientific (Waltham, MA, USA).

### 2.2. Microfluidic System Design and Fabrication

**Figure 1:**
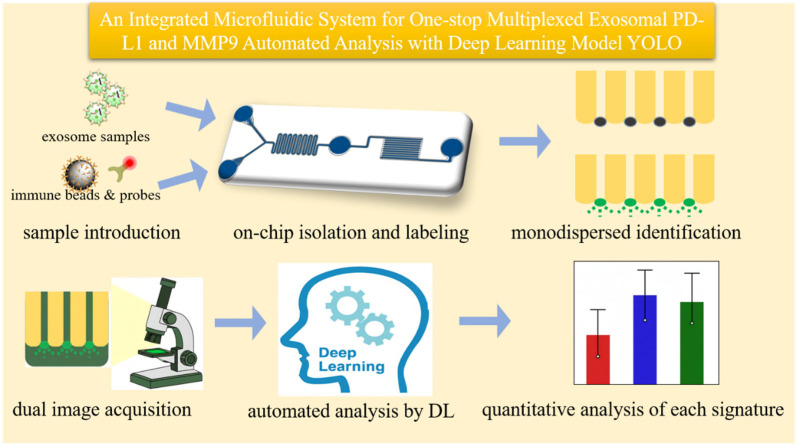
Schematic of the system principle. The diagram describes the overall design of the integrated microfluidic chip and the on-chip “sample-in, result-out” workflow for automated analysis.

The PDMS microchip was fabricated using standard soft lithography protocols. The chip layout was designed in AutoCAD 2018, with detailed dimensions available in the provided Supplementary CAD File, and transferred to a chromium photomask. A key feature of the design is a symmetric Y-type dual-inlet that converges into a single serpentine mixing channel and connected with subsequent micropillar arrays, as depicted in the device schematic (Figure 1). This channel is engineered to increase mix efficiency, ensuring rapid and homogenous mixing of co-injected sample and reagent streams before they enter a downstream circular incubation chamber. The analysis region consists of an array of micropillars precisely engineered for single-bead trapping. The pattern was used to create a master mold on a standard 4-inch (100 mm) silicon wafer via photolithography and deep reactive ion etching (RIE). To create an anti-adhesive surface for PDMS molding, the silicon master was silanized by vapor-phase deposition of PFOTS for 12 h under vacuum. A PDMS prepolymer and curing agent mixture (10:1 *w*/*w* ratio) was poured over the master, degassed in a vacuum chamber, and cured at 95 °C for 2 h. After curing, inlet and outlet ports were punched through the PDMS slab. The structured PDMS layer and a borosilicate glass coverslip (76 mm × 54 mm, Corning, NY, USA) were then treated with air plasma (Harrick Plasma, PDC-002, Ithaca, NY, USA) for 45 s at 180 W before being irreversibly bonded to form the final microfluidic device.

The analysis region consists of an array of micropillars precisely engineered for single-bead trapping (as shown in Supplementary Figure S1, with full dimensions available in the provided CAD File). The array is composed of 10 parallel rows, each with 60 micropillars, creating 59 individual traps per row for a total theoretical capacity of 590 monodispersed beads. To ensure all beads are retained for analysis, interception pillars are integrated at the outlet of the array, guaranteeing a 100% bead capture efficiency (demonstrated in Supplementary Figure S2d).

### 2.3. Preparation of Anti-CD63 Immuno-Magnetic Beads

Magnetic beads were functionalized with anti-CD63 capture antibodies to facilitate specific exosome isolation. The process was initiated by suspending 10 µL of carboxylic-functionalized beads in 90 µL of PBS, followed by two washing cycles via centrifugation. For carboxyl group activation, the beads were incubated in a solution containing 10 mg/mL Sulfo-NHS and 10 mg/mL EDC for 2 h at 25 °C under acidic conditions (pH 5.0). Following activation, the beads were pelleted and re-suspended in a 100 µL PBS solution containing the anti-CD63 capture antibody, followed by incubation for 2 h at 37 °C to facilitate covalent amide bond formation. A final wash cycle removed unbound antibodies, and the conjugated beads were resuspended in 100 µL of a blocking buffer (PBS-TB: 0.1% BSA and 0.02% Tween-20 in PBS) and stored at 4 °C until use.

### 2.4. Cell Culture and Supernatant Preparation

The HepG2, Cal-27, SK-LU-1, and HUVEC cell lines were confirmed to be mycoplasma-free and maintained in a Forma direct-heat CO_2_ incubator (Thermo Fisher Scientific, Waltham, MA, USA) at 37 °C with 5% CO_2_. The HepG2 and Cal-27 cell lines were cultured in DMEM. The SK-LU-1 cell line was cultured in MEM supplemented with 1% non-essential amino acids and 1% sodium pyruvate. All culture media were supplemented with 10% (*v*/*v*) FBS.

### 2.5. Isolation of Model Exosome Samples

For validation and characterization experiments, exosomes were isolated from cell culture supernatant by ultracentrifugation. Upon reaching 80% confluence, cultured cells were switched to a serum-free medium. The conditioned medium (~50 mL) was collected after 48 h. The isolation procedure involved sequential centrifugation at 4 °C: first at 2000× *g* for 30 min to pellet cells, then at 10,000× *g* for 30 min to remove larger debris. The resulting supernatant was then subjected to ultracentrifugation at 100,000× *g* for 70 min. This high-speed pelleting step was repeated once to ensure high purity. The final exosome pellet was resuspended in 100 µL of sterile PBS.

### 2.6. On-Chip Immunoassay Procedure

The entire immunoassay was performed on-chip. First, the microfluidic channels were primed by flowing PBS at a rate of 5 µL/min for 5 min to ensure all surfaces were wetted and to remove any trapped air bubbles. The exosome sample (cell culture supernatant) was injected through one inlet of the Y-junction, while a reagent cocktail was simultaneously injected through the other inlet at an equal flow rate of 2 µL/min for 5 min. This cocktail contained the anti-CD63 magnetic beads and the fluorophore detection probe for PD-L1 and MMP9. The serpentine channel design ensured rapid and efficient mixing of the two streams, initiating the formation of the bead–exosome–probe complexes. The mixture then flowed into the circular incubation chamber, where an external magnet was then placed on the chip for a 30 min incubation, which maintained the beads before the washing step (Supplementary Figure S2a). The beads were dispersed throughout the medium (Supplementary Figure S2b).

Following this, a washing buffer (PBST: PBS with 0.05% Tween-20) was introduced at 5 µL/min for 5 min while the external magnet remained in place to flush out unbound reagents. After washing, the magnet was removed, and the purified immuno-complexes were directed downstream into the micropillar array, where they were individually captured and immobilized for imaging (Supplementary Figure S2c). The beads are directed into the analysis array under a ‘controlled flow’. This term specifically refers to the oblique pressure and velocity fields generated by the unique geometry of the array. As demonstrated by the simulation results in Supplementary Figure S3a,b, this engineered flow profile is critical for achieving the high-efficiency, monodispersed capture of the beads, which is essential for accurate, single-bead level quantification.

### 2.7. Image Acquisition and Automated Data Analysis

Following on-chip capture, the micropillar array containing the immobilized immuno-complexes was imaged. Bright-field and fluorescence micrographs were acquired on an inverted fluorescence microscope using a 20× objective lens. U-MWIB2 filter sets were used for the fluorophore 488 signals of the PD-L1 and MMP9 probes. To ensure comparability across experiments, the exposure time was set at 1.2 s and black balance was 1200 consistently.

Quantitative analysis was performed using a custom-trained lightweight YOLOv5 deep learning model. The YOLO (You Only Look Once) architecture is a state-of-the-art object detection algorithm renowned for its balance of speed and accuracy. Its network structure is typically composed of three key parts: a “backbone” for extracting features from the input image at various scales, a “neck” that fuses these features to enrich the contextual information, and a “head” that performs the final detection to predict the location and class of objects. For this study, the model was specifically trained on a dataset of approximately 2000 microbead micrographs, which were manually annotated using LabelImg to create bounding boxes. The dataset was partitioned into training, validation, and test sets to develop a robust model tailored for microbead identification.

To ensure unbiased quantification and eliminate errors arising from low-signal or non-fluorescent beads, a positional migration strategy was implemented. This automated workflow operates in two distinct steps. First, the algorithm processes the high-contrast bright-field micrograph to accurately identify the location and delineate the precise boundaries (defined by coordinates x, y, w, h) of each individual microbead. This approach is highly accurate as it relies on the consistent physical features of the beads rather than variable fluorescence signals. Second, these positional coordinates are programmatically transferred, or “migrated,” to the corresponding fluorescence image. The mean grayscale intensity is then calculated exclusively from the round boundaries of these pre-defined beads. This two-step process decouples bead identification from signal intensity, enhancing accuracy and sensitivity by ensuring that all captured beads, regardless of their fluorescence level, are included in the analysis. This prevents the analytical bias that would occur if only fluorescently positive beads were detected and measured, a critical factor for samples with low target expression or high background noise.

## 3. Results and Discussion

### 3.1. Principle of the Integrated On-Chip Analysis

**Figure 2:**
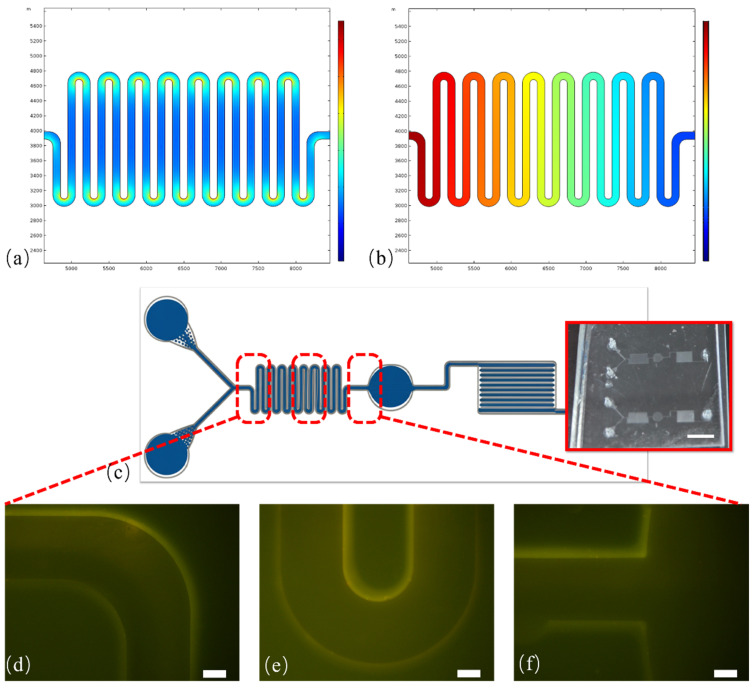
Simulation of the chip’s serpentine region and observation of on-chip mixing. COMSOL simulation of the velocity field (**a**) and pressure field (**b**) in the serpentine region. (**c**) Graph of the overall microfluidic chip, insert: the photo of prototype microfluidic chip, scale bar: 1 cm. (**d**–**f**) Progression of the mixing effect, where nano-sized fluorescent microspheres simulating exosomes are shown from the inlet, through the serpentine region, becoming uniformly dispersed before entering the incubation chamber, scale bar: 50 µm.

The operational workflow of the microfluidic system is designed for an integrated “sample-in, result-out” analysis, as depicted schematically in Figure 1. This “one-stop” approach signifies that the entire analytical process—from initial sample and reagent mixing, through exosome isolation and fluorescent labeling, to the final immobilization and detection—is performed sequentially within a single, self-contained chip. This integrated design eliminates the need for manual sample transfer between steps, significantly enhancing processing efficiency and minimizing sample handling. This integrated design eliminates the need for manual sample transfer between steps, significantly enhancing processing efficiency and minimizing off-chip sample handling. A Y-type configuration converges into a serpentine microchannel to induce efficient and rapid on-chip mixing of concurrently injected fluids. This feature allows for the simultaneous introduction of the biological sample through one inlet and a complex reagent mixture—containing anti-CD63-conjugated magnetic beads and the fluorophore-labeled anti-PD-L1/anti-MMP9 detection probe—through the other. The on-chip process begins with the co-injection of these streams. As they travel through the winding channel, they are thoroughly mixed to initiate binding events for 30 min. The efficiency of this mixing was validated using both COMSOL 5.3 simulations of the velocity and pressure fields and experimentally by tracking the dispersion of fluorescent nano-sized microspheres, which showed a transition from a laminar flow at the inlet to a homogenous mixture before the incubation chamber (Figure 2). After incubation and washing, the purified immuno-complexes are directed to an analysis region consisting of an array of precisely engineered micropillars.

The immobilization of beads within the analysis region is achieved through a sophisticated physical trapping mechanism. The design of the micropillar array generates both lateral and longitudinal gradient flow fields, which actively promote the guidance of microbeads into the traps (Supplementary Figure S3a,b). A key feature of this design is that once a trap is filled with a bead, the local longitudinal flow is obstructed, while the lateral flow component is maintained. This phenomenon directs the following beads to adjacent, empty traps, facilitating a sequential and high-efficiency loading process that ensures monodispersion. Under controlled flow, the beads are individually captured and immobilized, enabling single-bead resolution for subsequent analysis. This integrated design significantly enhances processing efficiency and minimizes sample handling.

### 3.2. Characterization of Isolated Exosomes

**Figure 3:**
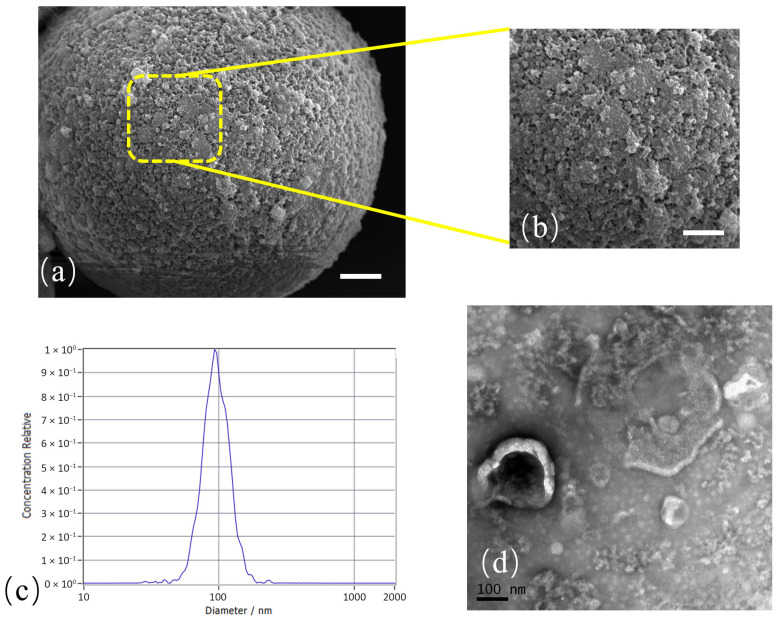
Characterization of exosomes. (**a**) Results of exosome capture on magnetic beads, scale bar: 1 µm. (**b**) A close-up view of a magnetic bead with captured exosome particles, scale bar: 800 nm. (**c**) Nanoparticle Tracking Analysis (NTA) result, showing a single peak at approximately 100 nm. (**d**) Transmission Electron Microscopy (TEM) image showing the typical cup-shaped morphology of exosomes.

Exosomes isolated from cell culture supernatant were first characterized to confirm their identity. The successful capture of exosomes onto anti-CD63 functionalized magnetic beads was visually confirmed (Figure 3a,b). Nanoparticle Tracking Analysis (NTA) revealed a particle size distribution with a distinct peak around 100 nm, consistent with the expected size range of exosomes, demonstrated the effective and purity isolation of exosomes (Figure 3c). Transmission Electron Microscopy (TEM) further validated the presence of vesicles exhibiting the classic cup-shaped morphology characteristic of exosomes (Figure 3d). These results collectively confirmed the successful isolation of a high-purity exosome population for subsequent experiments.

### 3.3. System Optimization and Analytical Performance

**Figure 4:**
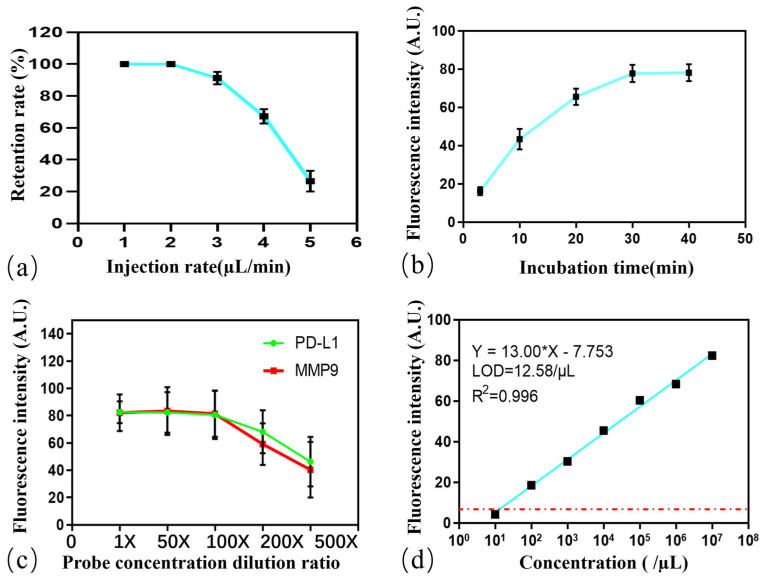
System parameter optimization. (**a**) Effect of injection flow rate on magnetic bead retention rate, showing complete retention at 1–2 µL/min. (**b**) Relationship between incubation time and fluorescence intensity, indicating signal saturation at 30 min. (**c**) Analysis of detection probe dilution ratios, with the 100× dilution of two probes selected as optimal. (**d**) Determination of the limit of detection (LOD) via serial dilution, the blue line indicates the linear fitting gradient dilution concentration curve, while the red dot line represents the fluorescence intensity value of the mean plus three times the standard deviation, showing high stability over a wide linear range (R^2^ = 0.996) and an LOD of 12.58 particles/µL.

To maximize the analytical performance of the microfluidic system, key operational parameters were systematically optimized. The efficiency of bead capture and retention in the analysis array is highly dependent on the injection flow velocity. Capture efficiency remained near 100% at flow rates of 1 and 2 µL/min but declined sharply at higher velocities. Therefore, 2 µL/min was selected as the optimal flow rate (Figure 4a). The incubation time required for the formation of the bead–exosome–probe complex was also investigated. We observed that the fluorescence signal for both markers rapidly increased and reached a plateau within approximately 30 min (Figure 4b). Extending the incubation beyond this point yielded no significant improvement in signal intensity, indicating that the immunoreactions had reached near-saturation. Finally, the concentration of the detection probes was titrated to achieve a maximal signal-to-noise ratio, with 100 dilution ratio being optimal for both the anti-MMP9 and anti-PD-L1 probe (Figure 4c).

The limit of detection (LOD) of the optimized system was determined by analyzing a serial dilution of exosomes derived from HepG-2 cell line supernatant. The exosome concentration, as quantified by Nanoparticle Tracking Analysis (NTA), spanned a range from 10 to 10^7^ particles/µL. A strong linear correlation (R^2^ = 0.996) was observed between the fluorescence intensity and the exosome concentration across this dynamic range. Based on the signal-to-noise ratio at the lowest concentrations, with the cell culture medium serving as a blank control, the LOD was calculated to be 12.58 particles per microliter (Figure 4d). This high sensitivity is attributed to the combination of efficient on-chip processing, the high signal-to-noise ratio afforded by the in situ detected fluorescent probes, and the precision of the automated YOLOv5-based quantification algorithm.

### 3.4. Automated Data Quantification Workflow

**Figure 5:**
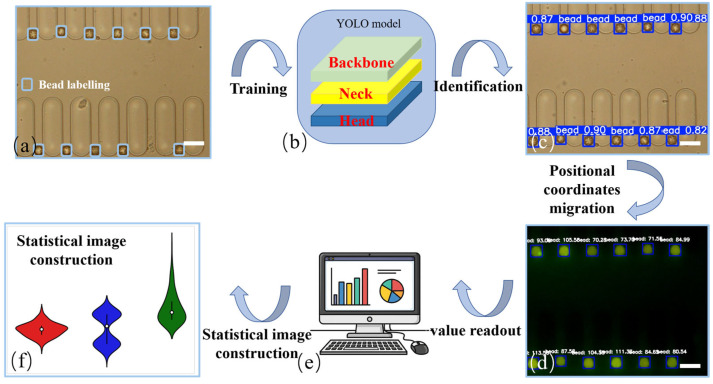
Workflow of the YOLOv5 model for automated quantification. (**a**) Example of manual annotation of magnetic beads within the micropillar gaps for training. (**b**) Schematic of the hierarchical structure of the YOLOv5 model, comprising the backbone, neck, and head. (**c**) Effective identification of each magnetic bead with a confidence score of about 0.9. (**d**) The positional migration strategy, where bead locations from the bright-field image are transferred to the fluorescence image for direct grayscale value reading. (**e**) Use of the model for automated statistical data analysis. (**f**) Output of the statistical results. Scale bar of (**a**,**c**,**d**): 30 µm.

To achieve high-throughput, unbiased and easy-to-handle data analysis for an untrained medical physician, a lightweight YOLOv5-based deep learning model was developed and implemented. The workflow for this automated quantification is outlined in Figure 5. The process begins with the manual annotation of microbeads in bright-field images to train the model (Figure 5a). The model employs a hierarchical structure of a backbone, neck, and head to efficiently process images (Figure 5b). In the identification stage, the trained model effectively recognizes each microbead with a high degree of confidence (~0.9) (Figure 5c). A key feature of the workflow is a positional migration strategy: the locations of all beads identified in the bright-field image are transferred to the corresponding fluorescence image, where the mean grayscale intensity is calculated for each bead (Figure 5d). This ensures that all beads, including non-fluorescent ones, are included in the analysis, preventing bias. The model then performs statistical analysis on the collected intensity data (Figure 5e) and outputs the final quantitative results (Figure 5f).

### 3.5. Multiplexed Profiling of Exosomal Markers Across Cancer Cell Lines

**Figure 6:**
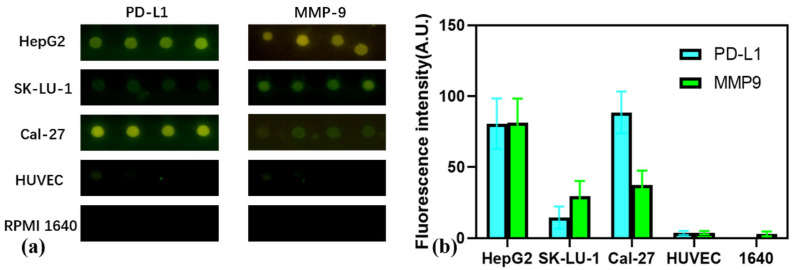
Multiplexed detection results for exosomal markers. (**a**) Representative fluorescence images from the multiplexed analysis of exosomes from different cell lines (HepG2, SK-LU-1, Cal-27), a HUVEC negative control, and an RPMI 1640 blank control. (**b**) A quantitative bar chart summarizing the analysis results for all samples.

To demonstrate the platform’s utility in differential diagnostics, a multiplexed assay was performed to profile exosomal PD-L1 and MMP9 expression across three distinct cancer cell lines: HepG2 (hepatocellular carcinoma), SK-LU-1 (non-small cell lung cancer), and Cal-27 (oral squamous cell carcinoma). As shown in the representative fluorescence micrographs in Figure 6a, a pronounced variation in protein expression was evident among the cell lines, with the HUVEC negative control and RPMI 1640 blank control showing negligible background signal

The quantified data, presented in Figure 6b, reveals unique and distinguishing protein signatures for each cell line. Exosomes derived from the Cal-27 cell line exhibited the highest level of PD-L1 expression, coupled with a moderate MMP9 signal. Conversely, HepG2-derived exosomes displayed high levels of both PD-L1 and MMP9. The SK-LU-1 cell line showed the lowest expression level for PD-L1 among the cancer lines, alongside a moderate MMP9 signal. This successful differentiation underscores the system’s capacity to generate specific proteomic fingerprints from complex biological samples, highlighting its potential utility in classifying tumor subtypes and elucidating their biological characteristics.

### 3.6. Discussion

The multiplexed analysis conducted using our integrated microfluidic platform successfully revealed distinct exosomal biomarker signatures for each of the investigated cancer cell lines. The assay showed that HepG2 exosomes exhibited a high-PD-L1/high-MMP9 profile; SK-LU-1 had a low-PD-L1 but high-MMP9 signature; and Cal-27 displayed the highest PD-L1 levels with moderate-to-high MMP9. These molecular fingerprints provide further insights together with existing reports. For instance, the high-PD-L1/high-MMP9 signature for the Cal-27 oral squamous cell carcinoma line is in strong agreement with the literature, confirming the platform’s accuracy. The high exosomal MMP9 detection for HepG2 supports more recent findings linking the protein to HCC progression, which may have been missed by traditional analysis methods. Furthermore, the intriguing divergence between SK-LU-1’s known high cellular PD-L1 expression and its low exosomal levels measured by our chip, which may be attributed to the high MMP9 activity in SK-LU-1 cells, which can cleave and shed surface PD-L1 as a soluble protein, thereby preventing its packaging into exosomes, highlights the system’s ability to provide unique biological information specific to the exosomal compartment. In sharp contrast to these cancer-specific profiles, the non-cancerous endothelial cell line HUVEC, used as a negative control, showed negligible levels of both exosomal markers. The clear distinction between the signals from the cancer-derived exosomes and the HUVEC control validates the platform’s specificity and ability to reliably distinguish cancerous from non-cancerous exosomal profiles. These clearly resolved molecular fingerprints underscore the system’s potential for differential diagnostics. Overall, these findings showcase the utility of our on-chip approach in capturing the multifaceted nature of cancer biology, presenting it as a potential tool for future tumor diagnostics and subtyping.

## 4. Conclusions

In this work, we have validated a one-stop integrated microfluidic system for the rapid, sensitive, and automated multiplexed analysis of exosomal protein markers. The dual-inlet Y-type architecture with a serpentine mixing channel enables efficient on-chip immunoreactions, while a subsequent micropillar array immobilizes immuno-complexes at a single-bead resolution, eliminating signal interference. This integrated design reduces the entire analytical workflow from sample introduction to result acquisition to under 30 min. The platform demonstrated a high degree of sensitivity, achieving a limit of detection of 12.58 particles/µL. Furthermore, the integration of a lightweight YOLOv5-based deep learning model with a positional migration strategy ensures accurate, easy-to-handle, and high-throughput data quantification, removing the potential for human bias.

Our successful application of the system to differentially profile PD-L1 and MMP9 expression on exosomes from three distinct cancer cell lines and a non-cancerous control highlights its potential as a powerful tool for cancer diagnostics. The ability to generate molecular fingerprints from small sample volumes could aid in tumor subtyping, provide valuable insights into the dominant mechanisms of tumor progression, and guide personalized therapeutic strategies. While this study successfully demonstrated the system’s capabilities using cell line-derived exosomes, we acknowledge that its performance has yet to be validated with more complex clinical samples, such as patient plasma. Future work will therefore focus on adapting the workflow for direct analysis of clinical samples and expanding the platform’s architecture to enable parallel processing of multiple samples. In summary, this microfluidic system represents a tangible step towards the realization of rapid, convenient, and efficient point-of-care liquid biopsies for cancer and other diseases.

## Data Availability

The source code for the modified YOLOv5 model are openly available in a public Hugging Face repository at https://huggingface.co/stardust202501/YOLO-based-exosome-analysis-system (accessed on 15 October 2025).

## Supplementary Materials

The following supporting information can be downloaded at: https://www.mdpi.com/article/10.3390/mi16111208/s1, Figure S1: Detailed design images of the microfluidic chip, Figure S2: Visualization of the on-chip bead incubation and magnetic maintain process, Figure S3: COMSOL simulation of the fluid dynamics within the detection array, File S1: One-stop integrated microfluidic system, Video S1: YOLO-based-exosome-analysis-system.
