# Supplementary material for: An Integrated Microfluidic System for One-Stop Multiplexed Exosomal PD-L1 and MMP9 Automated Analysis with Deep Learning Model YOLO
Source: Micromachines (Basel). 2025 Oct 24;16(11):1208. doi: 10.3390/mi16111208 (PMC12654492; doi:10.3390/mi16111208)
Supplement: Supplementary file 1 [file micromachines-16-01208-s001.zip › Data and Code Availability.pdf]

Data and Code Availability

The source code for the modified YOLOv5 model are openly available in a public Hugging Face repository at https://huggingface.co/stardust202501/YOLO-based-exosome-analysis-system
