# Supplementary material for: An Integrated Microfluidic System for One-Stop Multiplexed Exosomal PD-L1 and MMP9 Automated Analysis with Deep Learning Model YOLO
Source: Micromachines (Basel). 2025 Oct 24;16(11):1208. doi: 10.3390/mi16111208 (PMC12654492; doi:10.3390/mi16111208)
Supplement: Supplementary file 1 [file micromachines-16-01208-s001.zip › micromachines-3897150-supplementary.pdf]

Supplementary Material

An Integrated Microfluidic System for One-Stop Multiplexed Exosomal PD-L1 and MMP9 Automated Analysis with Deep Learning Model YOLO

Yunxing Lu [1,†], Wenjing Zhang [2,†], Qiang Shi [1], Jianan Hui [3,4], Jieyu Wang [3,5], Yiman Song [3,5] and Xiaoyue Yang [6,7,*]

[1] School of Science and Technology, Shanghai Open University, Shanghai 200433, China;
yunxinglu2025@163.com (Y.L.); shiqiang@sou.edu.cn (Q.S.)

[2] Department of Obstetrics and Gynecology, The Second Affiliated Hospital of Soochow University,
Suzhou 215000, China; zwj20190805@163.com

[3] State Key Laboratory of Transducer Technology, Shanghai Institute of Microsystem and Information
Technology, Chinese Academy of Sciences, Shanghai 200050, China;
jiananhui2@mail.sim.ac.cn (J.H.);
wjywaka@foxmail.com (J.W.); yimansong2025@163.com (Y.S.)

[4] Shanghai Frontier Innovation Research Institute, Shanghai 201108, China

[5] School of Stomatology, Dalian Medical University, Dalian 116044, China

[6] The International Peace Maternity and Child Health Hospital, School of Medicine, Shanghai Jiao Tong
University, Shanghai 200030, China

[7] Shanghai Key Laboratory of Embryo Original Diseases, Shanghai 200030, China

* Correspondence: yangxiaoyue@sjtu.edu.cn

Figure. S1. Detailed design images of the microfluidic chip.

Figure. S2. Visualization of the on-chip bead incubation and magnetic maintain process.

Figure S3: COMSOL simulation of the fluid dynamics within the detection array.

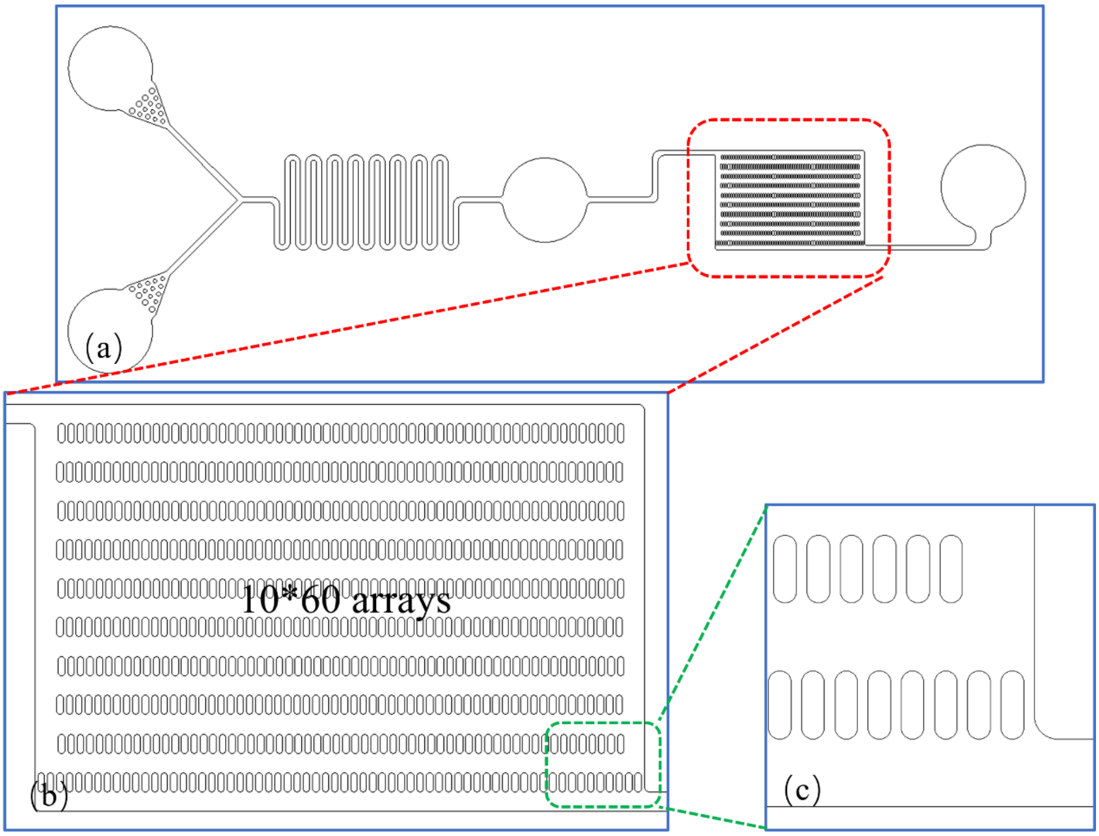

Figure S1: Detailed design images of the microfluidic chip. (a) ovall diagram of the microfluidic system. Detailed dimensions are available in the provided CAD file. (b) A magnified micrograph of the micropillar array designed for single-bead capture, showing the parallel row configuration capable of trapping up to 590 beads. (c) A micrograph highlighting the interception pillars located at the outlet of the analysis region, which eliminating the potential of sample loss.

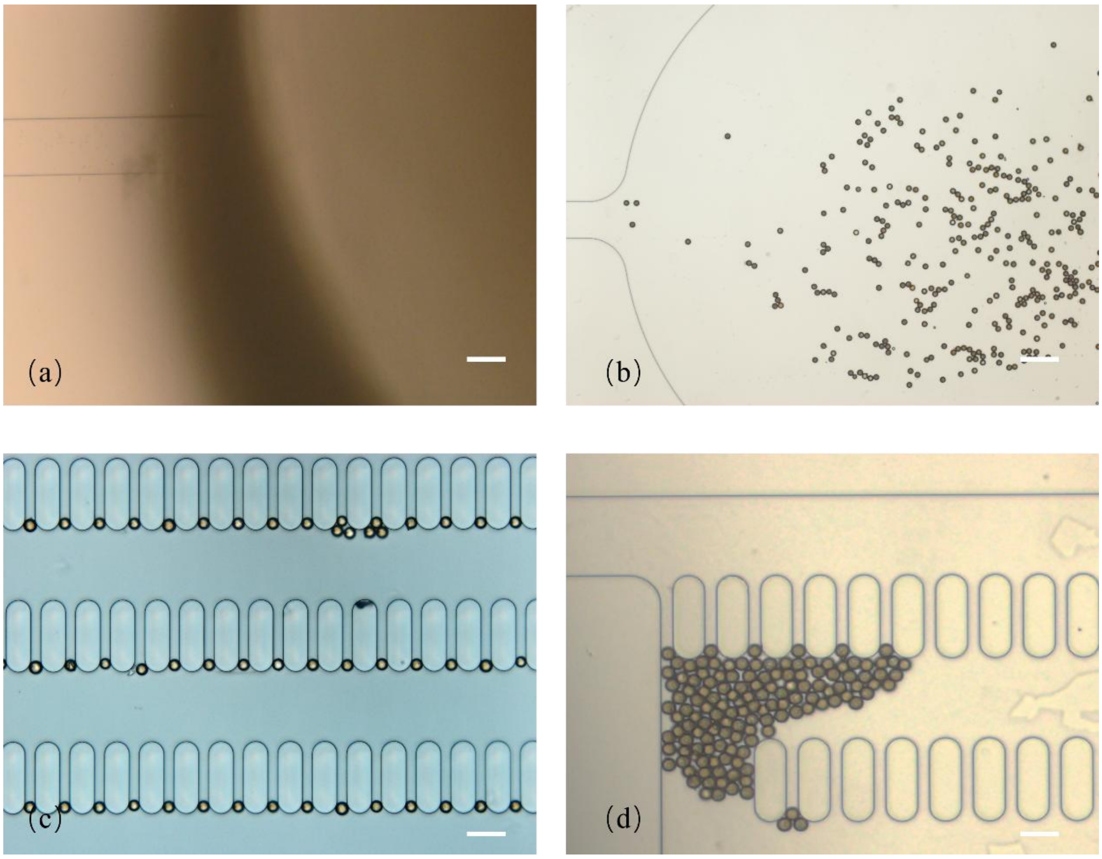

Figure S2: Visualization of the on-chip bead incubation and magnetic maintain process. (a) Micrograph showing the placement of an external magnet on the chip (position indicated by the shaded area on the right, scale bar: 100μm), where immuno-magnetic beads were aggregated within the incubation chamber. This demonstrates the efficient on-chip magnetic purification step. (b) Beads are shown dispersed throughout the chamber medium during the incubation phase, prior to magnetic aggregation and introduction into the analysis array via liquid flow, the magnet was temporarily removed for microphotography, scale bar: 100μm. (c) A representative image from the analysis region showing beads individually captured in the micropillar traps, achieving a monodispersed effect, scale bar: 50μm. (d) A micrograph of the interception pillars at the outlet, which physically block beads to ensure 100% capture efficiency, scale bar: 30μm.

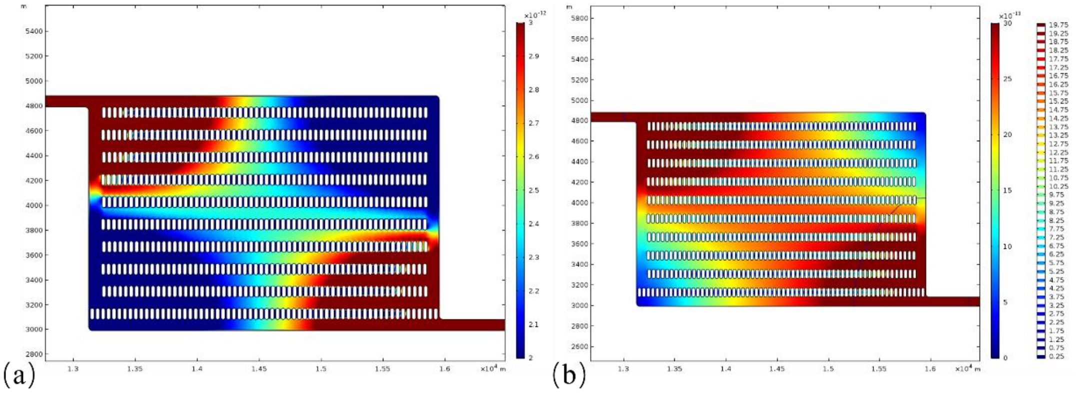

Figure S3: COMSOL simulation of the fluid dynamics within the detection array. (a) Simulation of the velocity field, showing the oblique flow profile at the inlet and outlet that constitutes the 'controlled flow'. (b) Simulation of the pressure field, demonstrating the pressure gradient that guides the microbeads into the traps. These engineered fields are responsible for the efficient, monodispersed bead capture observed in the experiments.
